# The progress of the microbe-gut-brain axis in sepsis-associated encephalopathy

**DOI:** 10.3389/fcimb.2025.1587463

**Published:** 2025-05-13

**Authors:** Chen He, Hui Shi, Zhijie Yu, Chunhan Ma, Zhiqiang Jiao, Jin Li, Fei Yang

**Affiliations:** ^1^ Chifeng Clinical Medical College of Inner Mongolia Medical University, Hohhot, China; ^2^ Department of Critical Care Medicine, Chifeng Municipal Hospital, Chifeng, China; ^3^ Department of Emergency Medicine, Chifeng Municipal Hospital, Chifeng, China

**Keywords:** sepsis-associated encephalopathy, microbe-gut-brain axis, gut microbiota, neuroimmune, neuroendocrine

## Abstract

Sepsis-associated encephalopathy (SAE) is a diffuse brain dysfunction that is caused by sepsis without direct brain injury or central nervous system infection and is manifested as anxiety-like behavior and cognitive dysfunction. The microbiota-gut-brain axis, on the other hand, is a bidirectional communication network between the gut and the brain that modulates host behavior and cognitive function in many ways and is of central importance in the preservation of general health and homeostasis. Given the functional roles attributed to the microbiota-gut-brain axis (MGBA), contemporary research is progressively focused on elucidating relationships between SAE and alterations in compositional and quantitative intestinal microbiota profiles. This review consolidates interdisciplinary insights from immunology, microbiology, neuroendocrine signaling, and neural pathophysiology to evaluate the mechanistic contribution of the MGBA to the relief of cognitive impairments in SAE. By unifying these perspectives, with the aim of preventing or enhancing SAE-related neurological dysfunction for the formulation of MGBA-targeted therapeutic strategies.

## 1 Introduction

Sepsis is a life-threatening systemic disorder as a result of an unbalanced host response to infection that progresses to multi-organ dysfunction by means of pathological immune activation (Singer et al., 2016). SAE is one of the most significant complications of sepsis and occurs in 30% to 70% of sepsis patients (Han et al., 2023). Individuals with SAE commonly present with a spectrum of cognitive impairments, including impaired sustained attention, memory dysfunction, and spatial disorientation. These neuropsychological sequelae not only adversely affect the acute recovery phase but also compromise long-term functional independence and overall quality of life (Zong et al., 2019). Clinical investigations demonstrate that individuals with sepsis exhibit a significantly elevated mortality risk. This risk escalates two- to threefold in cases where SAE coexists (Chen et al., 2020; Ge et al., 2022). However, SAE arises from multifactorial interactions among diverse pathophysiological processes, including excessive glial cell activation, persistent neuroinflammatory activity, compromised blood-brain barrier (BBB) integrity, and edematous alterations in vascular and cellular structures. These mechanisms collectively disrupt neural homeostasis, contributing to SAE progression (Centner et al., 2024). The precise molecular mechanisms underlying cerebral injury in the context of sepsis remain poorly characterized. This knowledge gap has hindered the development of targeted therapeutic interventions in clinical practice.

The gastrointestinal system serves as a critical factor in the sepsis and multiple organ dysfunction syndrome (MODS), acting as a pivotal driver in critical illness. Mediating this relationship, the microbiota-gut-brain axis (MGBA) constitutes a bidirectional communication network that integrates intestinal activity with central nervous system functions. This axis modulates essential physiological processes including immune regulation, nutritional metabolism, and circadian rhythms through three primary mechanisms: microbial interactions, neurological pathways, and humoral signaling mechanisms (Qian et al., 2023). Disruption of gut microbiota in SAE not only induces dysregulated MGBA signaling and promotes heightened production of pro-inflammatory mediators but also impairs critical processes associated with neurotransmitter synthesis, degradation, and blood-brain barrier permeability (Yan et al., 2023). This series of alterations has the potential to exacerbate SAE. The currently employed strategies for managing SAE, based on gut flora regulation by MGBA, have been proven to be of great value. Probiotic supplementation, colony transplantation, and other novel therapeutic approaches are among the potential interventions that have been explored. This review will focus on the research progress of MGBA in the field of SAE, with the objective of providing a new theoretical basis and research reference for in-depth investigation of the pathogenesis and therapeutic approaches of SAE.

## 2 Gut microbiota alterations in SAE

Under physiological conditions, the composition of the gut microbiota is characterized by stability and diversity. The protective functions of the microbiota extend even to the nervous system, where it regulates the differentiation of regulatory T cells and promotes anti-inflammatory responses through the production of specific metabolites. Additionally, the microbiota can regulate host’s mood and cognitive functions by stimulating the enteric nervous system (ENS). The ENS, in turn, produces various neurotransmitters, such as 5-hydroxytryptophan (5-HT) and glutamate, which interact with the cerebral cortex (Ragonnaud and Biragyn, 2021). The presence of gut microbes has been demonstrated to stimulate the secretion of glucagon-like peptide-1 (GLP-1) by endocrine cells. This, in turn, activates the vagus nerve within the intestine, thereby facilitating the transmission of signals to the brain. These signals influence appetite, metabolism, and, thus regulate energy balance and the psychological state (Zhang et al., 2022).

**Figure 1:**
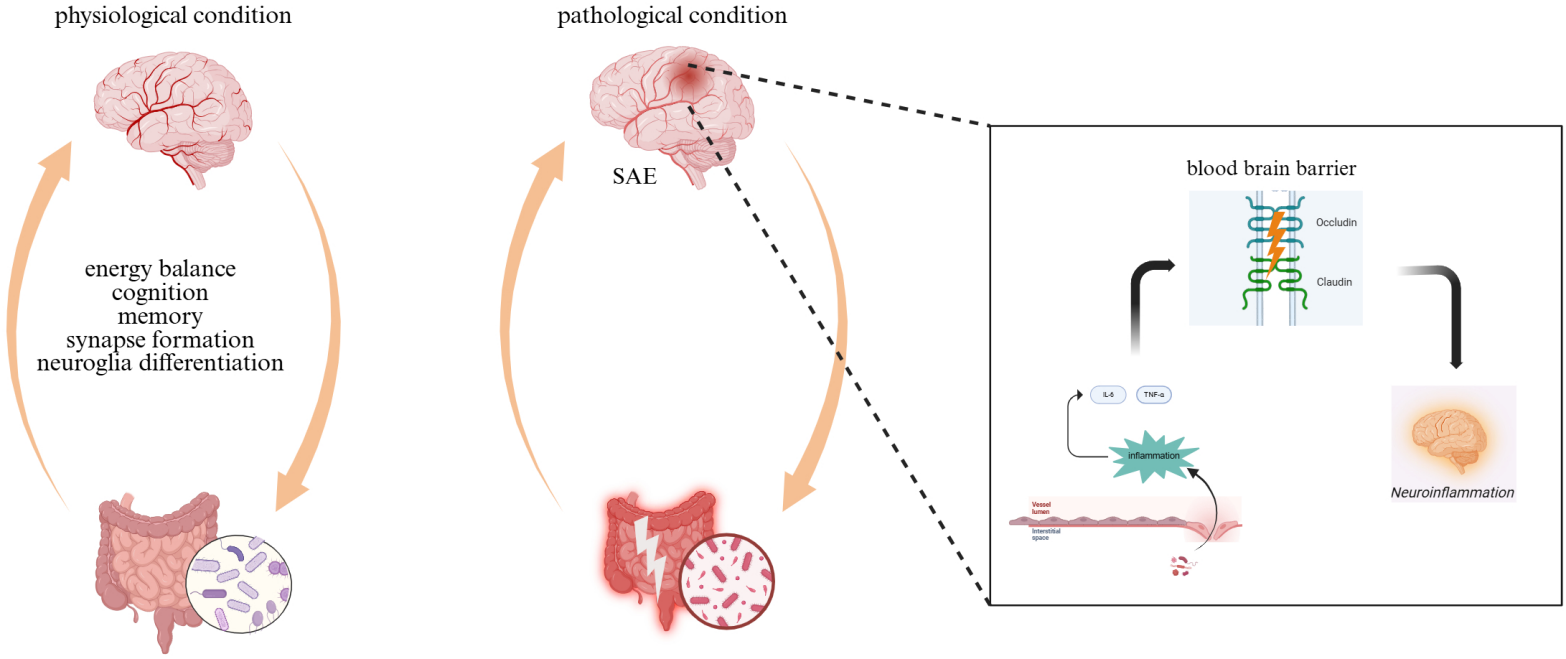
Different states of the microbe-gut-brain axis.

However, the diversity of gut microorganisms was significantly reduced and the community structure was markedly altered following damage to the gut barrier. For example, the abundance of the thick-walled phylum is significantly reduced, while the relative abundance of the facultative phylum increases. This affects the systemic metabolic and immune status of the host. During sepsis, the expression of tight junction proteins such as occludin and claudin is down-regulated and the intestinal barrier function is disrupted. This leads to a considerable decrease in butyric acid-producing bacteria, such as Rostridium spp. and Clostridium perfringens, and an increase in the number of pathogenic bacteria, such as Enterobacteriaceae. Subsequently, elevated levels of plasma lipopolysaccharides (LPS) emerge, inducing systemic inflammatory processes and triggering the secretion of numerous pro-inflammatory mediators such as interleukin-6 (IL-6) and tumor necrosis factor-α (TNF-α). These mediators traverse the BBB, leading to excessive activation of cerebral microglia and astrocytes, thereby instigating neuroinflammatory cascades. The compromised integrity of the BBB permits the passage of IL-6 and other inflammatory mediators into neural tissues, which further stimulates the hyperactivity of glial cells (Liu et al., 2022; Zhao and Jia, 2024). Pathogenic communication between systemic inflammatory signaling and immune activation within the CNS propagates chronic neuroinflammation. The chronic inflammatory process is a fundamental mechanism in SAE pathogenesis (**Figure 1**).

## 3 Mechanisms of action of MGBA in SAE

### 3.1 Neural pathway

The brainstem within the MGBA is integrally involved in processing mechanical and chemical signals transmitted via vagal afferent pathways. Research indicates that acetylcholine originating in the medullary regions interacts with α7 nicotinic acetylcholine receptors (α7-nAChR) expressed on enteric neurons, astrocytes, and microglial cells. This interaction modulates neuroinflammatory responses and synaptic plasticity mechanisms, thereby influencing higher-order neural functions including cognitive processing and memory formation. The vagus nerve primarily functions through the cholinergic anti-inflammatory pathway (CAP). By suppressing excessive inflammatory activity, significantly mitigates the likelihood of shock and multi-organ dysfunction linked to septic conditions (Pontes-Arruda et al., 2011). Extensive research demonstrates elevated levels of pro-inflammatory cytokines, including interleukin-1β (IL-1β), TNF-α, and IL-6, in murine sepsis models. Nevertheless, stimulation of the CAP via GTS-21 dihydrochloride has been observed to effectively mitigate the decline in these pro-inflammatory mediators (Xie et al., 2020). Prior research indicates that intraperitoneal injection of lipopolysaccharide (LPS) in murine models disrupts acetylcholine-mediated signaling pathways within hippocampal regions, potentially altering cognitive and behavioral functions associated with its activity. This decline was associated with aggravated deficits in neuronal activity and synaptic adaptability in the hippocampus, which subsequently precipitated the emergence of cognitive deficits. Cholinergic neurotransmission could be seen to mitigate the damage to neuronal function and synaptic plasticity in the hippocampus, thereby enhancing sepsis-induced cognitive dysfunction (Yin et al., 2023a). As noted, breakdown of neurotransmitters within the brain, specifically, lack of cholinergic transmission, triggers release of heterogeneous pro-inflammatory mediators and hyper-activation of microglia to amplify neural responses toward end accumulation of neuroinflammation along with cognitive impairment. Furthermore, the occurrence of aberrant alterations in the community of gut microbiota is also expected to have a direct influence on the brain through the vagus nerve and thus cause the induction of an inflammatory response of a systemic nature. Gut flora of healthy rats implanted into septic rats has been found to enhance learning and memory in the host rats. However, the same effect is disrupted when the vagus nerve is severed (Li et al., 2018). Besides, mice models exposed to lipopolysaccharide and subjected to subdiaphragmatic vagotomy (SDV) had elevated levels of TrkB/brain-derived neurotrophic factor (BDNF) expression in the hippocampus. This observation indicates that SDV effectively countered the LPS-induced alterations in intestinal microbiota and cognitive dysfunction (Basavaraju et al., 2023). It can be observed that the vagus nerve may influence the development of systemic inflammatory response by regulating gut flora and improving cognitive function. Further studies on the mechanism of vagus nerve activity in SAE have the potential to provide novel therapeutic strategies for its treatment.

**Figure 2:**
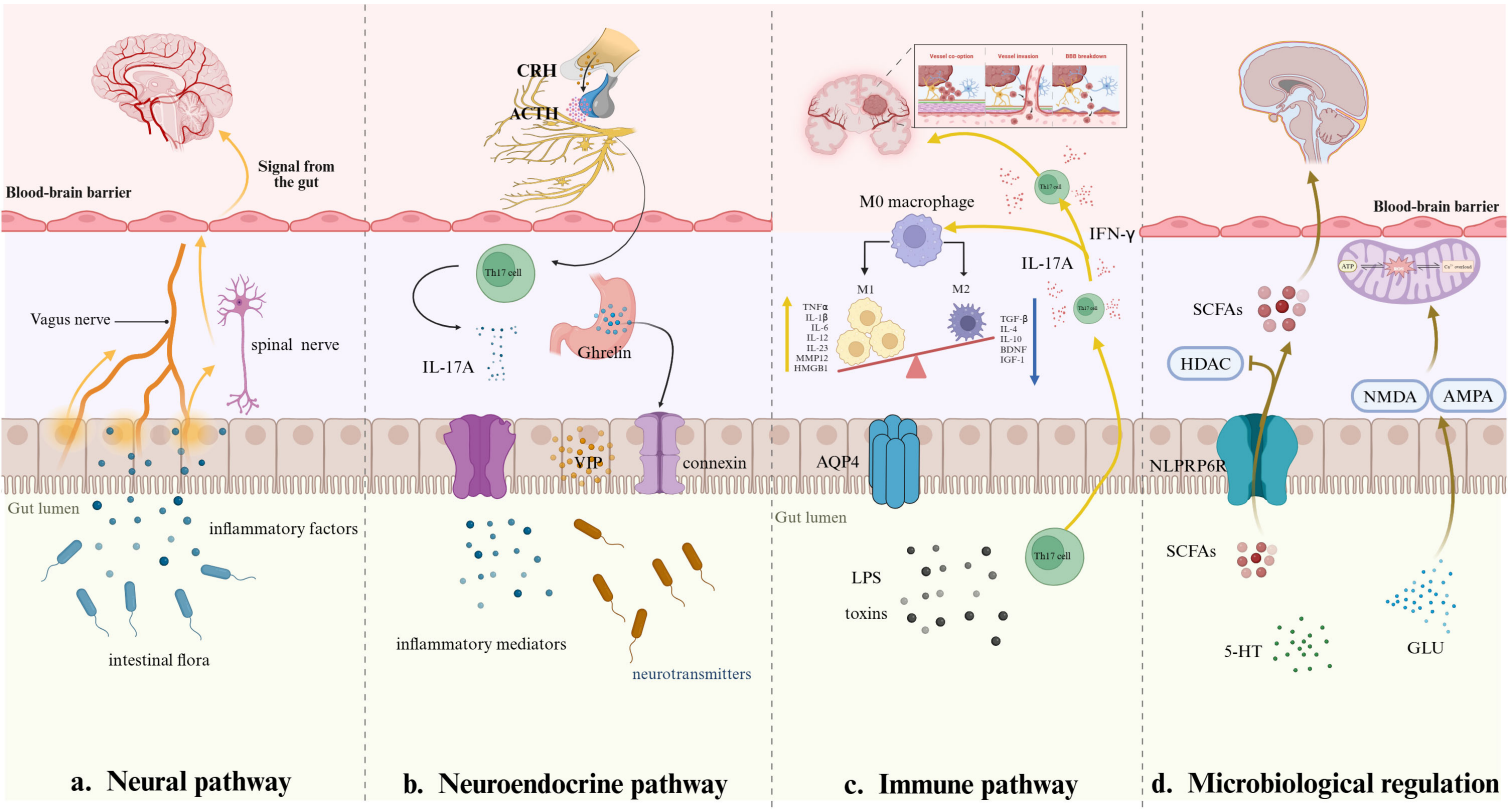
Four pathways through which the gut-brain axis transmits inflammatory signals.

Accordingly, stimulation in the intestinal tract can be transmitted via spinal afferent nerve fibers to specific regions of the brain, including the sensory cortex. During the septic state, inflammatory mediators released from peripheral tissues bind to corresponding receptors on spinal afferent nerve fibers, subsequently activating immune cells such as microglia and astrocytes within the brain. Consequently, neuronal dysfunction occurs, inducing the clinical manifestations characteristic of SAE (Chen et al., 2024). This pathway plays a significant role in maintaining cerebral metabolic homeostasis. During sepsis, systemic metabolic imbalances induce disrupted cerebrovascular regulation, impaired glucose utilization, and neuronal mitochondrial impairment (Xu et al., 2022). These lead neuronal injury, thereby facilitating the progression of SAE (**Figure 2a**).

### 3.2 Neuroendocrine pathways

#### 3.2.1 The hypothalamic-pituitary-adrenal axis

The hypothalamic-pituitary-adrenal (HPA) axis functions as a primary regulatory network for physiological stress adaptation, facilitating enhanced mobilization of energy reserves while curtailing non-vital metabolic processes to preserve critical physiological functions. Activation of this axis triggers elevated production and release of key endocrine signaling molecules, including corticotropin-releasing hormone (CRH), adrenocorticotropic hormone (ACTH), and cortisol. During the initial phases of sepsis, excessive activation of the HPA axis induces dysregulation in CRH, ACTH, and cortisol levels. This imbalance subsequently promotes neuroinflammatory processes, ultimately culminating in brain damage (Spencer-Segal et al., 2020). Specifically, CRH induces the synthesis of pro-inflammatory mediators and interacts with specific microglial receptors, thereby activating the TLR4/NF-κB signaling pathway. This activation facilitates microglial phenotypic polarization, which contributes to the modulation of neuroinflammatory processes. ACTH interacts with melanocortin receptors within the CNS, suppressing immune cell activity and producing anti-inflammatory outcomes. Cortisol suppresses the production of pro-inflammatory mediators, such as cytokines and prostaglandin E2 (PGE2), while concurrently attenuating the recruitment and accumulation of polymorphonuclear leukocytes (PMNs). Furthermore, cortisol promotes the polarization of macrophages toward the M2c phenotype, facilitating a modulatory effect on inflammatory pathways and contributing to the resolution of immune overactivation (Sun et al., 2022; Yu et al., 2023). Experimental data indicated that hydrocortisone administration in septic mice led to decreased plasma ACTH concentrations and reduced expression of CRH mRNA (Téblick et al., 2022). These neuroendocrine changes correlated with enhanced behavioral outcomes, suggesting a potential therapeutic effect on neurological function during sepsis. Furthermore, glucocorticoids have been shown to stimulate the HPA axis, resulting in heightened intestinal permeability. Consequently, Th17 cells are induced to release interleukin 17A (IL-17A), initiating a series of events that provoke inflammatory reactions within both the gastrointestinal tract and the central nervous system (**Figure 2b**) (Yang et al., 2023). In summary, there is a strong correlation between the hyperactivation of the HPA axis and the development of two major pathologies: severe immunosuppression and imbalance in the regulation of intestinal homeostasis. These pathologic changes, in turn, lead to the development of SAE.

#### 3.2.2 Other neuroendocrine hormones

GLP-1 receptors are widely distributed throughout the central nervous system. During the initial phase of sepsis, heightened GLP-1 concentrations augment metabolic energy utilization as a physiological adaptation to infection, primarily through modulation of glucose homeostasis. However, as the condition advances, persistent systemic inflammation coupled with compromised intestinal barrier integrity disrupts GLP-1 secretory patterns or induces impairments in receptor signaling (Perl et al., 2018). These pathophysiological alterations exacerbate metabolic dysregulation, further complicating the host’s adaptive capacity. Research indicates that GLP-1 receptor activation has the capacity to suppress excessive microglial activity and curtail the production of pro-inflammatory mediators, mitigating neuroinflammation in SAE. Conversely, a reduction in beneficial gut microbiota may result in diminished GLP-1 production, potentially exacerbating SAE pathogenesis through dysregulated inflammatory pathways (Diz-Chaves et al., 2018; Li et al., 2018). This research looks at the effect of GLP-1 receptor agonists on intestinal toxins and systemic markers of inflammation. Findings are that these drugs effectively reduce the levels of toxic substances while improving cortical blood flow and inhibiting neuronal death (Reich and Hölscher, 2022). The protective effects on the nerve cells are correlated with reduced brain damage, as evidenced by better metrics of neural tissue integrity.

Ghrelin and vasoactive intestinal peptide (VIP) are neuropeptides with broad-ranging central nervous system influences. Experimental research indicates that ghrelin inhibits microglial and astrocytic cell activation, regulates neuroinflammatory signaling pathways, and increases levels of major neurotransmitters such as dopamine and acetylcholine. These concerted actions cumulatively increase neuronal excitability and promote synaptic plasticity, allowing adaptive neural network reconfiguration. This peptide hormone also stimulates intestinal epithelial cell proliferation and increases the expression of tight junction-associated proteins. Experiments show that gastrin maintains the intestinal barrier, reduces bacterial and endotoxin passage across the mucosal surface, and regulates neuroinflammatory phenomena by stimulating systemic anti-inflammatory mechanisms (Ishioh et al., 2020; Sarlaki et al., 2022). VIP, however, acts on CNS receptors, inhibiting microglial activation and regulating neuroinflammatory phenomena and cerebral blood flow patterns. Through this mechanism, VIP can produce neuroprotection, preventing SAE development. VIP, together with neuropeptide Y (NPY), suppresses inflammatory mediator release and increases neuronal tolerance to stress factors during the early phase of sepsis. As sepsis progresses, however, the sustained increase in NPY leads to metabolic derangements and neurological impairment (**Figure 2b**) (Li et al., 2019a; Bian et al., 2024). These findings indicate that VIP and NPY exhibit distinct functional roles during distinct phases of sepsis progression, modulating pathophysiological processes in a stage-dependent manner.

### 3.3 Immune pathway

#### 3.3.1 Intestinal immunity

Immune signaling molecules generated by intestinal immune cells exhibit bidirectional interactions with the gut microbiota. These bioactive compounds can permeate the BBB, subsequently stimulating CNS immune cells and triggering neuroinflammatory cascades (Bostick et al., 2022). During sepsis, the activation of the gut immune system instigates a series of physiological responses, including the release of various cytokines. These cytokines fulfill a dual role, acting to stimulate the release of inflammatory substances from cerebrovascular endothelial cells while concomitantly potentially compromising the integrity of the BBB (Pinitchun et al., 2024). Th17 cell generation and activity in intestinal settings play a bifunctional role: the cells induce local inflammation but also create immune homeostasis. In the course of sepsis development, Th17 cell populations relocate to the CNS, where they secrete pro-inflammatory mediators interferon-γ (IFN-γ) and IL-17A to induce microglial activation. The activated microglia then increase the production of interleukin-1β (IL-1β) and interleukin-23 (IL-23), which, in turn, augments IL-17A expression (**Figure 2c**) (Moraes et al., 2021). The cycle facilitates neuroinflammatory processes, worsening CNS pathology. Furthermore, IL-23 drives Th17 cell differentiation toward a pro-inflammatory phenotype that secretes GM-CSF+, IFN-γ+, and CXCR3+ subsets. This phenotypic alteration disrupts the harmony of neuroimmune interactions and imparts a significant dysregulation of inflammatory signaling pathways implicated in cerebral immune regulation (Gao et al., 2024b). The findings point to the pivotal role of Th17 lymphocytes in inflammatory disorders of the central nervous system, including SAE and cerebral ischemic injury. However, additional studies are warranted to elucidate their underlying molecular mechanisms and determine their therapeutic potential, subject to further empirical validation.

#### 3.3.2 Brain immunity

Microglia, the brain’s resident immune cells, exhibit two states of polarized activation, known as M1 and M2, which have opposing functional roles in neuroinflammation and neuroprotection. The M1 phenotype initiates inflammatory pathways by releasing mediators like high mobility group protein 1 (HMGB1) and matrix metalloproteinase 12 (MMP12), which enhance pro inflammatory signaling (He et al., 2023). Studies show that microglia in animal models of SAE exhibit long term activation associated with the HMGB1 dependent autoregulatory signaling pathway. This persistent hyperactivity induces pathological alterations in synaptic excitatory functional and structural properties and reductions in neuronal activity in the hippocampus. These dysfunctions progressively undermine neuroplasticity, causing cognitive impairments (Yin et al., 2023b). Conversely, the M2 phenotype is associated with anti-inflammatory and repair processes. M2-type microglia secrete brain-derived neurotrophic factor (BDNF), which acts via the regulation of synaptic plasticity. This not only reduces neuronal cell death in the hippocampus of septic mice, but also improves cognitive processes such as memory consolidation and learning (Choi et al., 2024). During the initial progression of sepsis, the CCL/CCR5 signaling axis facilitates the translocation of M1-polarized microglia and upregulates the connexin Claudin-5, which improves the stability of the BBB. However, persistent inflammatory activity shifts microglial polarization toward a phagocytic M2 phenotype, which destabilizes BBB integrity through structural degradation and compromised barrier permeability. This breakdown enables infiltration of harmful peripheral agents, including inflammatory mediators and neurotoxic compounds, into the CNS, inducing neuroinflammatory cascades (Lin et al., 2023; Yang et al., 2024). It has been demonstrated that the augmentation in the proportion of M1-type microglia, in conjunction with the imbalance between M1 and M2 polarization, results in an escalation of inflammatory mediator release. This, in turn, precipitates neuronal damage and neuroinflammation (**Figure 2c**).

Astrocytes, the most prevalent glial cells within the CNS, play a pivotal role in ensuring the maintenance of the BBB, a process that facilitates neuroprotection. As demonstrated by the research, the secretion of pro-inflammatory cytokines such as IL-1α and TNF-α by activated microglia in the context of brain injury results in the loss of function of type A1 astrocytes, thereby altering their role in normal astrocyte function. Emerging evidence suggests that non-hepatic hyperammonemia enhances aquaporin-4 (AQP4) expression in astrocytes via the gut-microbiota-brain axis (MGBA), inducing astrocyte edema, decreasing cerebral blood flow, and causing neuronal damage, leading to worsening of SAE (Zhao et al., 2024). Further studies indicates that AQP4 exacerbates cognitive impairments linked to sepsis by suppressing astrocyte-mediated autophagy and impairing anti-inflammatory functions. This alteration is mainly regulated by molecular signaling pathways involving peroxisome proliferation-activated receptor (PPAR) and mammalian target of rapamycin (mTOR) systems (Zhu et al., 2023). Recent findings indicate a link between sepsis and intestinal microbial dysbiosis, coupled with decreased levels of microbial-derived metabolites such as indole propionic acid (IPA). And, IPA suppresses NLRP3 inflammasome activation in microglial cells and attenuates LPS-induced IL-1β release through mechanisms dependent on the aryl hydrocarbon receptor (AhR). This intervention disrupts neuroinflammatory signaling cascades, thereby mitigating inflammation-mediated damage in neural tissues (Fang et al., 2022). This interplay suggests a plausible pathway through which intestinal microbiota may mediate immune regulation and influence neuroinflammatory processes via modifications in neuronal activity.

### 3.4 Microbiological regulation

#### 3.4.1 Short-chain fatty acids (SCFAs)

Short-chain fatty acids (SCFAs), synthesized through microbial fermentation of dietary fibers in the gut, play a vital role in preserving the structural and functional integrity of the gut epithelial barrier while regulating immune system equilibrium. Beyond their local gastrointestinal roles, these metabolites influence neurotransmitter activity and traverse the blood-brain barrier. Within the central nervous system, SCFAs upregulate tight junction protein synthesis in brain regions like the frontal cortex and hippocampus, reducing vascular permeability in neural tissues and ameliorates cognitive impairments linked to inflammatory-driven neurodegenerative or metabolic disorders (Zhang et al., 2023b). However, sepsis-induced gut microbiota dysbiosis correlates with diminished SCFAs levels, contributing to astrocyte proliferation within the frontal cortex and hippocampal regions. Such elevations in astrocytic activity are further implicated in the induction of neurocognitive impairments (Zhang et al., 2023b). Emerging evidence suggests that SCFAs modulate neuroinflammatory pathways by stimulating NLRP6 inflammasomes within the colon. These compounds enhance hippocampal neurogenesis, evidenced by elevated doublecortin-positive (DCX^+^) neuronal populations, while concurrently suppressing neuroinflammatory markers in hippocampal tissues. Such mechanisms are further implicated in attenuating systemic neuroinflammation associated with SAE (**Figure 2d**) (Li et al., 2019b). Regarding the regulation of oxidative stress, it has been shown that butyrate activates the Nrf2 signaling pathway in response to oxidative stress by inhibiting histone deacetylase (HDAC) (Jang et al., 2021). This is extremely critical to attenuate inflammation and cellular damage in septic states. In terms of disease regulation, SCFAs not only play an anti-inflammatory role by inhibiting HDAC and activating some G-protein-coupled receptors (GPCRs) on the cell surface, but also inhibit inflammation by inhibiting the overactivation of midbrain nigrostriatal microglia through their metabolites, such as sodium butyrate, which restores their function and induces phenotypic transformation (Zhang et al., 2023a). Thus, SCFAs are associated with sepsis intestinal flora disorders, which impact various physiological functions. This association offers novel mechanistic approaches to the treatment of sepsis.

#### 3.4.2 Neurotransmitter precursors and metabolites

Tryptophan is a precursor of 5-HT and kynurenine. Inflammatory responses can activate indoleamine 2,3-dioxygenase (IDO), resulting in a massive conversion of tryptophan to kynurenine. This may be associated with an exacerbation of SAE-related neuroinflammation. In the Alzheimer’s disease mouse model, IDO inhibitors reduced kynurenine production, significantly attenuated microglia activation and neuroinflammation in the brain, and ameliorated cognitive dysfunction in mice (Lewerenz and Maher, 2015). Furthermore, quinolinic acid, a metabolite of kynurenine, has been shown to possess neurotoxic properties. In the context of SAE, quinolinic acid levels have been observed to exhibit a marked increase, which subsequently activates N-methyl-D-aspartate (NMDA) receptors. This activation results in an accumulation of intra-neuronal calcium, leading to neuronal damage and death (Lee et al., 2024). Subsequent research demonstrated a pathological association between depressive-like behaviors and cognitive impairments in patients with SAE, correlating with reduced 5-HT concentrations. Experimental interventions involving tryptophan supplementation or pharmacological regulation of its metabolic pathways were associated with amelioration of neuropsychiatric manifestations, suggesting a mechanistic link between 5-HT homeostasis and neurological outcomes in SAE (Zhang et al., 2024). However, further research is needed to investigate the pathophysiologic mechanisms of how tryptophan affects SAE and to find possible points of intervention to mitigate the long-term effects of sepsis on brain function.

In neurodegenerative diseases, excessive amounts of glutamine overstimulate NMDA and α-amino-3-hydroxy-5-methyl-4-isoxazolepropionic acid (AMPA) receptors. Overstimulation of receptors by these receptors initiates a series of detrimental cellular mechanisms, including excessive intracellular calcium, mitochondrial damage, and enhanced oxidative stress within the neurons. These processes slowly compromise neuronal integrity, both structural stability and functional competence, and culminate in neuronal apoptosis (**Figure 2d**) (Huo et al., 2021). There is recent evidence implicating that disruption of glutamine homeostasis can contribute to the pathogenesis of sepsis. Manipulation of glutamine metabolism—through pharmacologic regulation of glutamine synthetase, enzymatic modulation, or administration of synthetic analogs—can be neuroprotective, reducing neuroinflammatory processes and preserving neurological function during systemic inflammation (Revuelta et al., 2020). This represents an appealing therapeutic alternative to manage sepsis-associated cognitive impairments. Glutamate is also a precursor of γ-aminobutyric acid (GABA) through enzymatic conversion by glutamic acid decarboxylase (GAD). In SAE, a pronounced reduction in GAD activity is observed, concurrent with a substantial upregulation of GABA transaminase (GABA-T). This dysregulation disrupts GABA homeostasis, causing diminished cerebral GABA concentrations. Consequently, heightened neuronal excitability ensues, precipitating excitotoxic neuronal injury. Notably, pharmacological activation of GABA receptors through agonist administration demonstrates neuroprotective efficacy, attenuating such damage (Gao et al., 2024a).

## 4 MGBA-based treatment of sepsis-associated-encephalopathy

### 4.1 Probiotics

Probiotic interventions are now widely integrated into clinical protocols, with Lactobacillus spp. and Bifidobacterium spp. frequently utilized as therapeutic agents. These microorganisms exert modulatory effects on host immunity and neurological function by restoring equilibrium within the intestinal microbial ecosystem. Notably, Bifidobacteria demonstrate pronounced efficacy in optimizing the structural composition of enteric microbiota. Experimental studies using SAE mouse models treated with Bifidobacterium strains revealed a notable elevation in the proportional representation of commensal bacteria within the gastrointestinal tract. This shift triggered a marked attenuation of intestinal inflammatory activity, thereby establishing bidirectional communication between gut homeostasis and neuroinflammatory pathways, which correlated with reduced neuroinflammatory pathology (Dong et al., 2022). Furthermore, research indicates that Bifidobacteria enhance ZO-1 protein synthesis within the intestinal epithelium. These microorganisms also diminish gut barrier permeability, thereby inhibiting the translocation of pathogenic agents and endotoxins into systemic circulation (Kim et al., 2022). Such mechanisms effectively mitigate the risk of initiating a systemic inflammatory cascade. Research indicates that the presence of Lactobacillus spp. in the gut microenvironment stimulates the growth and functional engagement of immune cell subsets, particularly regulatory T cells (Tregs), in conditions associated with SAE. Moreover, these bacteria secrete essential immunomodulatory molecules, including interleukin-10 (IL-10), which has a primary role in the inhibition of excessive inflammatory responses via the regulation of harmful cytokine signaling pathways (Chen et al., 2022). Lactobacilli indirectly influence tryptophan metabolism via intestinal microbial metabolite interactions, thereby modulating neurochemical levels such as 5-HT. Regulation of this neurotransmitter has been linked to improvement in spatial navigation, memory consolidation, and cognition performance metrics (Gao et al., 2020).

### 4.2 Fecal mushroom transplantation

Fecal microbiota transplantation (FMT) has been shown to be an effective novel therapeutic approach to SAE. In preclinical studies, SAE-carrying mice that received FMT from healthy donors exhibited gut microbiota structures very similar to donor patterns, with significant restoration of microbial diversity and enrichment of beneficial bacteria such as Lactobacillus and Bifidobacterium. In addition, FMT treatment was correlated with diminished levels of systemic pro-inflammatory cytokines such as TNF-α and IL-6. These immunological changes were paralleled by enhanced motor function and exploratory behavior in treated animals, suggesting alleviation of SAE-mediated neurological dysfunction (Gai et al., 2021). The findings can be mechanistically accounted for by FMT-induced upregulation of tight junction proteins in recipient animals’ intestines. Improvement in such barrier integrity limits the translocation of enteric bacteria and endotoxins into systemic circulation and their dissemination into peripheral tissues. Clinical investigations also demonstrate that selective FMT administration in selected populations of SAE patients is associated with measurable improvement of cognitive function and executive control, as corroborated by standardized neurobehavioral testing (Hazan, 2020). Nonetheless, additional studies are required to determine the therapeutic applicability of FMT in treating SAE. This is due to the fact that the variability realized in donor microbial compositions and methodological heterogeneity in treatment regimen approaches might undermine the strength of therapeutic consistency. To surmount these shortcomings, strictly controlled clinical trials are necessary for determining the most effective paradigms of administration and facilitating reproducible clinical findings.

### 4.3 Targeted therapies

In the clinic, glucocorticoid receptor (GR) antagonists have emerged as a promising therapeutic approach in the treatment of a variety of pathological disorders. SAE patients who received GR antagonist therapy exhibit reduced neuroinflammatory markers, a benefit in addition to the well-characterized cognitive improvement of attentional capacity and memory retention, which is provided by this pharmacologic treatment. Experimental research using murine models also demonstrates that pharmacologic disruption of glucocorticoid signaling pathways resulted in profound reduction of anxiety-related phenotypes with normalization of monoaminergic neurotransmitter systems, including norepinephrine and dopamine levels (Karen et al., 2021). Clinical research reveals that adrenocorticotropin-releasing hormone receptor antagonists can possibly increase patient survival and reduce the occurrence of complications when properly used.

## 5 Conclusion

Our comprehensive research on the mechanism of action of MGBA in SAE has offered new insights into the pathologic mechanisms and interventional strategies of SAE. These involve multi-level signaling of neural, endocrine, immune, and metabolic regulation, and the ecological balance between the host and microorganisms. Recent studies have elucidated that gut dysbiosis in SAE drives systemic inflammatory responses and penetrates the blood-brain barrier by releasing pathogen-associated molecules and metabolite disorders, ultimately leading to neuroinflammation and brain function damage. However, the evolution of gut flora structure and function during different periods of sepsis may have differential regulatory effects on specific cells such as microglia and astrocytes in the brain. This suggests that our intervention strategies need to be tailored to the progression of the disease. For instance, the initial phase is characterized by the suppression of systemic inflammatory storms, while the subsequent phase is marked by the emphasis on ecological restoration of the colony and neuro-regeneration. Moreover, metabolites have the potential to influence the long-term prognosis of brain function, underscoring the significance of prompt intervention.

Current therapeutic strategies targeting MGBA show some potential, but their clinical application still faces the dual challenges of precision and safety. Therapies such as probiotics and FMT have been shown to improve cognitive function in animal models. However, the efficacy of these therapies has been variable in clinical studies due to strain heterogeneity, individual tolerance differences, and differences in the host intestinal microenvironment. For instance, severe disruption of gut ecology in critically ill patients may limit the colonization efficiency of FMT, while over-supplementation of probiotics may raise the risk of flora imbalance. Consequently, microbial therapy or specific modulation based on key nodes of host-flora interactions may become a safer and more controllable direction. Concurrently, the screening of biomarkers (e.g., flora signature profiles, metabolite combinations, and inflammatory factors) in conjunction with multi-omics technology can facilitate the early diagnosis and subtyping classification of SAE, thereby providing a foundation for individualized treatment regimens.

Consequently, research in the domain of the microbe-gut-brain axis is precipitating a paradigm shift in the study of SAE toward a theoretical framework referred to as “systems regulation theory.” This shift is of significant importance, as it not only directs our attention toward investigating the mechanisms of MGBA in sepsis-associated encephalopathy but also provides a crucial reference point for the research on other neuroimmune diseases. The targeting of MGBA to enhance SAE is not only associated with the treatment of sepsis-associated encephalopathy, but also provides a valuable reference point for the study of the mechanisms underlying other neuroimmune diseases.
